# Bleeding complications in cholecystectomy: a register study of over 22 000 cholecystectomies in Finland

**DOI:** 10.1186/s12893-015-0085-2

**Published:** 2015-08-13

**Authors:** S. Suuronen, A. Kivivuori, J. Tuimala, H. Paajanen

**Affiliations:** Department of Surgery, Mikkeli Central Hospital, 50100 Mikkeli, Finland; Finnish Red Cross Blood Service, 00100 Helsinki, Finland; Department of Surgery, Kuopio University Hospital, PL 1777, 70600 Kuopio, Finland; School of Medicine, University of Eastern Finland, 70600 Kuopio, Finland

**Keywords:** Laparoscopic cholecystectomy, Open cholecystectomy, Bleeding complication

## Abstract

**Background:**

Major bleeding is rare but among the most serious complications of laparoscopic surgery. Still very little is known on bleeding complications and related blood component use in laparoscopic cholecystectomy (LC). The aim of this study is to compare bleeding complications, transfusion rates and related costs between LC and open cholecystectomy (OC).

**Methods:**

Data concerning LCs and OCs and related blood component use between 2002 and 2007 were collected from existing computerized medical records (Finnish Red Cross Register) of ten Finnish hospital districts.

**Results:**

Register data included 17175 LCs and 4942 OCs. In the LC group, 1.3 % of the patients received red blood cell (RBC) transfusion compared to 13 % of the patients in the OC group (*p* < 0.001). Similarly, the proportions of patients with platelet (0.1 % vs. 1.2 %, *p* < 0.001) and fresh frozen plasma (FFP) products (0.5 % vs. 5.8 %) transfusions were respectively higher in the OC group than in the LC group. The mean transfused dose of RBCs, PTLs and FFP product Octaplas® or the mean cost of the transfused blood components did not differ significantly between the LC and OC groups.

**Conclusions:**

Laparoscopic cholecystectomy was associated with lower transfusion rates of blood components compared to open surgery. The severity of bleeding complications may not differ substantially between LC and OC.

## Background

During the last two decades laparoscopic cholecystectomy (LC) has become the golden standard of the treatment of symptomatic gallbladder disease. Compared to the traditional open cholecystectomy (OC), LC is associated with lower morbidity [1] and mortality [2], shorter length of hospital stay and faster return to normal activities [3]. However LC is associated with higher incidence of iatrogenic bile duct injuries than OC [4–6].

Still, according to register studies, some 10 to 30 % of all cholecystectomies are performed using open technique, particularly in elderly population [7] and in acute cholecystitis [2, 8]. In addition, the open technique is still needed, when the laparoscopic operation cannot be completed safely and the conversion to open procedure is required. According to the literature, current conversion rate varies between 5 and 10 % [9–12]. The majority of conversions are performed because of obscure anatomy (difficult cholecystitis) or bleeding complications.

The incidence of bleeding complications requiring transfusion or reoperation has been reported to be relatively rare, occurring in 0.1 % in patients undergoing LC [13]. Focus in the literature, however, has been on biliary complications of LC. Yet, major vascular complications, even though rare, are also serious complications of laparoscopy [14, 15]. In addition, bleeding remains a frequent reason for conversion [9, 10, 16, 17]. Regarding OC, only few studies have reported the incidence of bleeding complications in the laparoscopic era. Bleeding has been reported to occur in 0.4 % of patients undergoing OC [4]. There are very little data on transfusion rates of red blood cells (RBCs) and hospital costs related to bleeding in cholecystectomies.

The purpose of this study is to compare transfusion rates, amounts of transfusions and related costs between LC and OC in a large Finnish register-based cohort. No prior data of blood transfusion and related costs in LC is currently available.

## Methods

**Figure 1:**
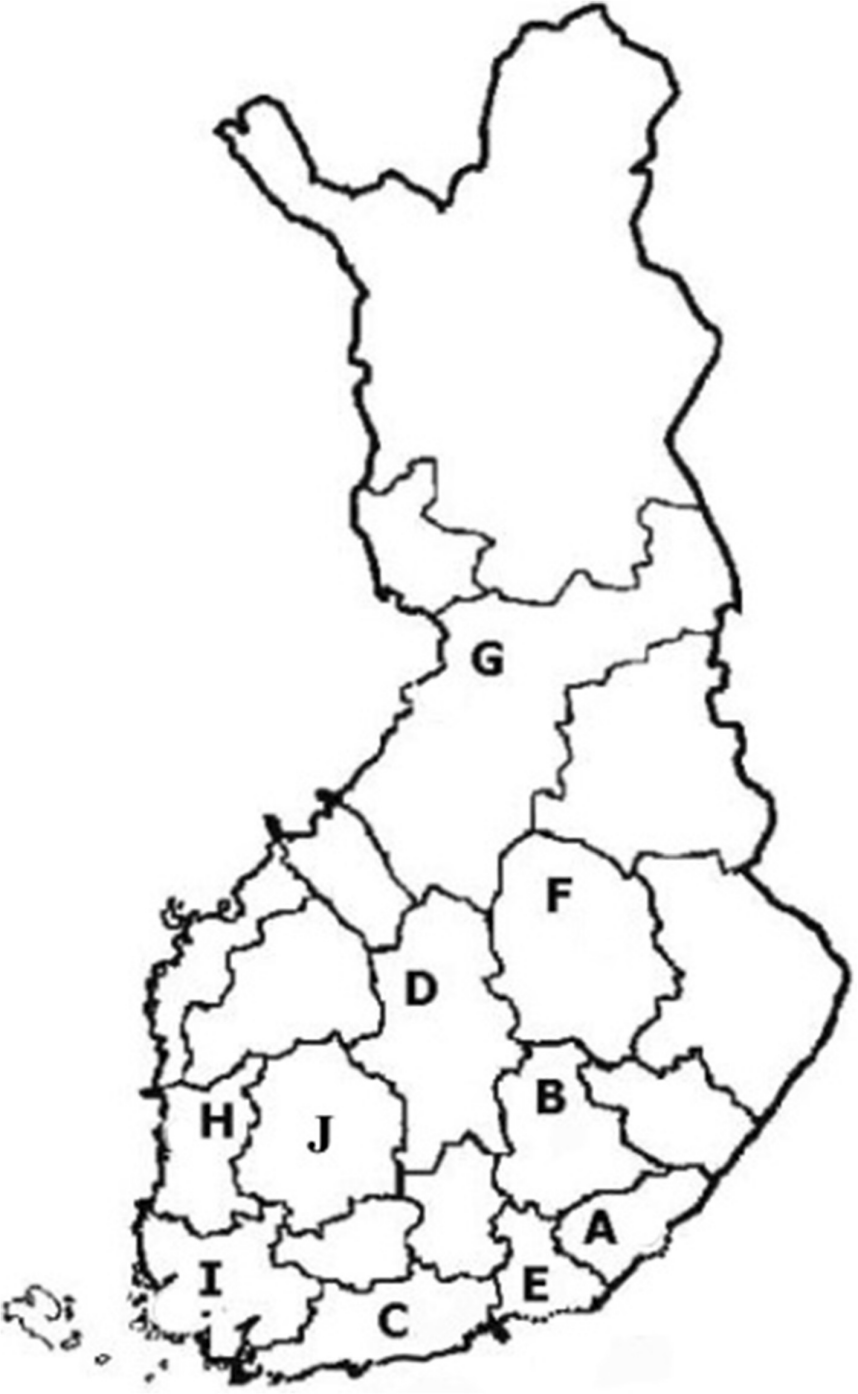
Participated Finnish hospital districts

Data from potentially transfused patients was collected to a separate “Optimal Use of Blood” (VOK) registry in a joint effort between the Finnish Red Cross Blood Service and ten (out of 21) Finnish hospital districts. Five of the hospital districts were teaching university-connected hospital districts (C, F, G, I and J) and five central hospital districts (A, B, D, E and H) (Fig. 1). The bleeding registry was started in 2002 and continually updated between 2002 and 2011, but the registry has been permanently terminated and erased in 2012. The data collection system is described in more details by Palo and co-workers [18]. Data were collected from hospitals which fulfilled the technical prerequisites and could deliver the required data. Altogether these hospital districts have about 620 000 inpatient episodes annually, constituting 63 % of all Finnish public inpatient hospital episodes. Patient data came from pre-existing electronic medical registers. Computer files provided information on hospital admissions, diagnoses, surgical operations, test results, and blood components as well as on transfusions. The originally collected 90 transfusion-related variables were processed and combined into 149 variables to describe an episode. Systematic audit of data variable was done [18].

For this study, patients who visited any study hospital district for LC or OC between January 1^st^ 2002 and December 31^st^ 2007 were extracted from the original VOK registry (2002–2011). In this research registry, data from the hospital district J is only available from Jan 1^st^ 2004 to December 31^st^ 2007.

ICD-10 (International Statistical Classification of Diseases and Related Health Problems, 10th Revision, 2003) was used to categorize diagnoses. Surgical procedure was defined according to the NCSP (The NOMESCO, Nordic Medico-Statistical Committee, Classification of Surgical Procedures) classification (NCSP–the NOMESCO Classification of Surgical Procedures version 1.7, 2005). All patients over 15 years of age who underwent LC (JKA21) or OC (JKA20) as a primary surgical procedure were included in this study. NCSP does not have a separate procedure code for conversion of LC converted into OC. Thus, conversions cannot be identified from this data and they are reported in the OC group. Cholecystectomies associated with biliary or pancreatic neoplasms were excluded based on the main diagnosis of the operation and diagnoses for the hospital admission. All procedure codes and diagnoses were set by treating physicians.

The following data was collected for each patient: age, sex, American Society of Anaesthesiologists (ASA) class, the main diagnosis, the primary surgical procedure, secondary surgical procedures, the status of the operation (either elective/prescheduled or emergent/acute case), the length of the operation, the usage of blood components during the hospital stay, in-hospital mortality, the length of hospital stay, the hospital, the hospital district and reoperations performed within 60 days of the cholecystectomy (not reported). Emergent operations were performed mostly because of acute cholecystitis or long-lasting painful biliary colic, which required hospital admission. The recorded usage of blood components included the number of transfused red blood cell (RBC) units, number of transfused platelets (PLT), number of transfused fresh frozen plasma products and the total cost of the transfused blood components. The severity of acute cholecystitis (flegmonous/perforated) was not included, because no detailed operation files or pathological reports from removed gallbladders were available.

The Finnish Red Cross Blood Service collects and distributes blood products from non-remunerated donors nationwide in Finland [19]. Each RBC product is equivalent of 1 unit. The PLT product was prepared using a buffy-coat procedure and most of the transfused PLTs were buffy-coat derived. The mean number of PLTs per four-donor product was 296 × 109 and the volume was about 320 ml [20]. Each PLT product is equivalent of (3-)4 units.

The fresh frozen plasma products consisted of fresh frozen plasma (FFP) and Octaplas® (Octapharma Nordic AB). The FFP refers to whole blood–recovered leukodepleted fresh plasma (volume, approximately 270 ml). One product was equal to 1 unit of FFP [21]. During the study period, Octaplas® was available in Finland from 2005 until the end of the study. The usage of the FFP and Octaplas® are presented separately in this study, because the unit size and thus the amount of coagulation factors per unit is smaller in Octaplas® compared to FFP.

The number of all cholecystectomies performed in Finland in 2000–2007 was obtained from the National Institute for Health and Welfare (NIHW) registry.

Permission to use the Bleeding registry data was obtained from the institutional review board of the Finnish Red Cross Blood Service. We did not use patients’ identification details (name or social security number) in this register study.

The aims and content of this study are in accordance with the Helsinki Declaration. The respective ethics committee of the Finnish Red Cross Blood Service approved the study protocol.

The data was analyzed using IBM SPSS Statistics 22.0 (IBM, USA 2015). The independence of two categorical variables was tested for with *χ*^2^ test. Mean values of continuous variables were compared with either Student’s *t*-test or Mann–Whitney *U* test where appropriate. Statistical significance was defined as a p value less than 0.05.

## Results

**Figure 2:**
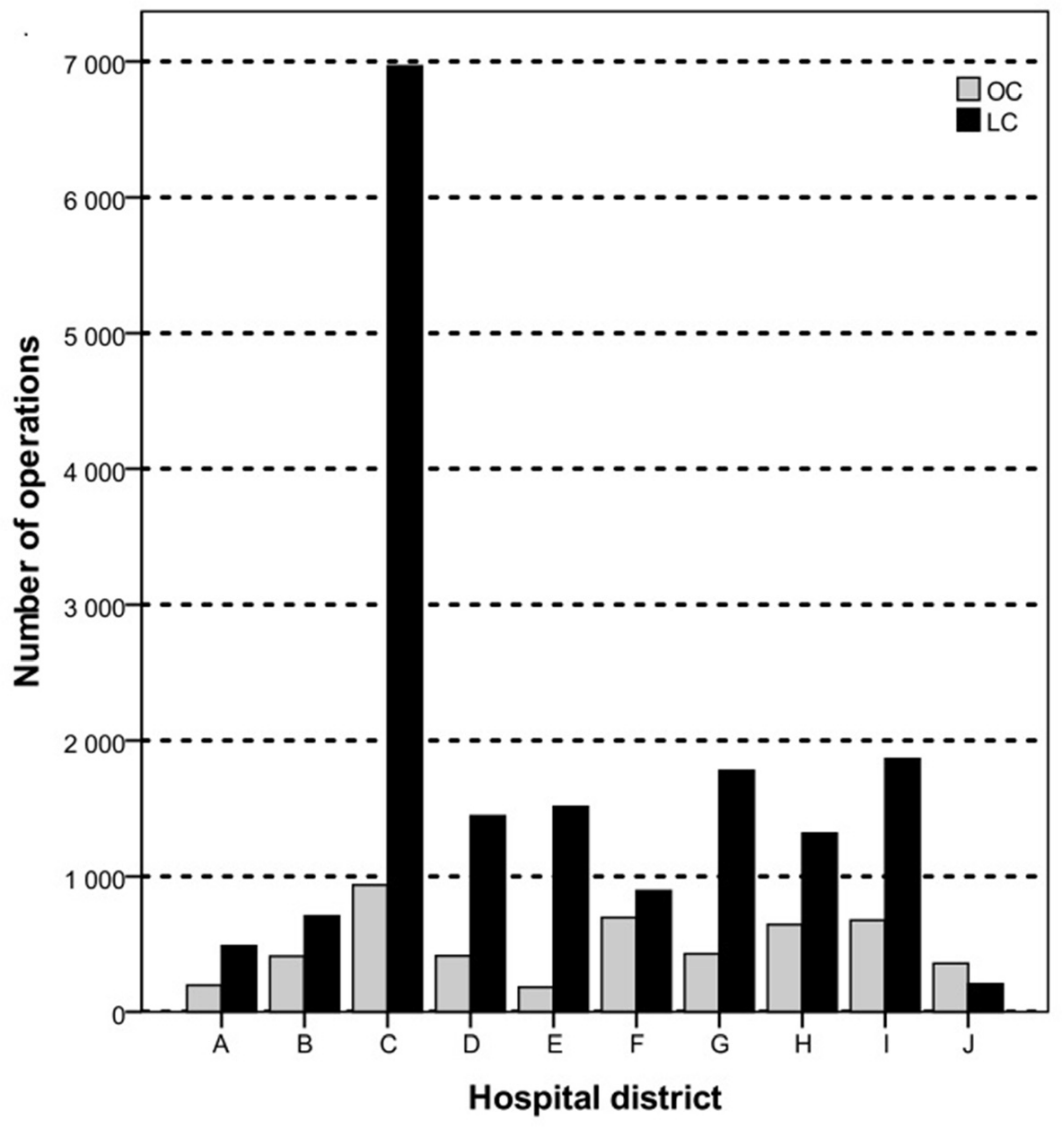
Number of laparoscopic cholecystectomies (LC) and open cholecystectomies (OC) by hospital districts in 2002 – 2007 (from hospital district J data available only from 2004 to 2007)

The total of 22 117 cholecystectomies comprise the final data set for this study accounting for 43 % all cholecystectomies (51 094) performed in Finland in 2002–2007. Of the included cholecystectomies, 78 % (17 175) were LCs and 22 % (4942) were OCs. The number of LCs and OCs by hospital district is shown in Fig. 2.

Demographic and perioperative data of the cholecystectomy patients is shown in Table 1. Compared to the OC patients, the LC patients were younger, more often female and more often belonged to the lower ASA classes. They also underwent an elective operation more often than the OC patients (88 % vs. 38 %, *p* < 0.001). The mean length of the operation was shorter for LCs than for OCs. Intraoperative cholangiographies (IOC) and common bile duct explorations were more common in the OC group. In addition, the mean length of hospital stay was longer and in-hospital mortality was higher among the OC patients (2.5 % vs. 0.3 %, *p* > 0.001).

**Table 1:** Demographic and perioperative data of patients who underwent open cholecystectomy (OC) or laparoscopic cholecystectomy (LC) in 2002 – 2007

	LC	OC	*p*
	*n* = 17175 (%)	*n* = 4942 (%)	
Males/females	4702/12473 (27/73)	2429/2513 (49/51)	<0.001
Mean age ± SD (range)	52 ± 15 (16 – 94)	63 ± 15 (16 – 97)	<0.001
ASA^a^ I	5842 (34.0)	673 (13.6)	<0.001
II	6424 (38.0)	1564 (31.6)	<0.001
III	2753 (16.0)	1756 (35.5)	<0.001
IV	183 (1.1)	409 (8.3)	<0.001
V	0 (0.0)	31 (0.6)	<0.001
Elective/emergency	15114/2018^b^ (88/12)	1870/3039^c^ (38/62)	<0.001
Mean operative time ± SD (min)	70 ± 37	99 ± 50	<0.001
Intraoperative cholangiography	862 (5.0)	1009 (20.0)	<0.001
Common bile duct exploration	156 (0.9)	369 (7.5)	<0.001
In-hospital mortality	59 (0.3)	122 (2.5)	<0.001
Length of hospital stay ± SD (d)	2.8 ± 2.4	8.0 ± 4.7	<0.001

^a^ *ASA* American Society of Anaesthesiologists, *SD* Standard deviation

^a^Data missing from 1974 patients in the LC group and 509 patients in the OC group

^b^Data missing from 43 patients

^c^Data missing from 33 patients

The blood component use associated with LC and OC is shown in Table 2. Of the OC patients, as many as 16 % of patients received transfusion of any blood component compared to 1.6 % of the patients in the LC group. Similarly, the proportions of patient with RBC (13 % vs. 1.3 %, *p* < 0.001), PLT (1.2 % vs. 0.1 %, *p* < 0.001), FFP (4.9 % vs. 0.4 %, *p* < 0.001) and Octaplas® (0.9 % vs. 0.1 %, *p* < 0.001) transfusions were respectively higher in OC group compared to the LC group. Also the mean transfused dose of the FFP was significantly higher in the OC group compared to the LC groups. However, the mean transfused dose of the other blood components and the mean cost of the transfused blood components did not differ significantly between the groups (Table 2).

**Table 2:** The use of blood components in open cholecystectomies (OC) and laparoscopic cholecystectomies (LC) in 2002–2007

	LC	OC	*p*
	*n* = 17175 (%)	*n* = 4942 (%)	
Proportion of patients with RBC transfusion	216 (1.3)	641 (13)	<0.001
Mean transfused RBC dose (range) (unit)	3.4 (1 – 18)	3.6 (1 – 46)	ns
Proportion of patients with PTL transfusion	15 (0.1)	59 (1.2)	<0.001
Mean transfused PTL dose (range) (unit)	16 (4 – 48)	21 (3 – 104)	ns
Proportion of patients with FFP transfusion	74 (0.4)	241 (4.9)	<0.001
Mean transfused FFP dose (range) (unit)	3.2 (1 – 10)	4.3 (1 – 42)	0.008
Proportion of patients with Octaplas® transfusion	14 (0.1)	43 (0.9)	<0.001
Mean transfused Octaplas® dose (range) (unit)	3.2 (1 – 12)	4.2 (1 – 15)	ns
Proportion of patients with blood component transfusion	276 (1.6)	774 (16)	<0.001
Mean cost of transfused blood components (range) (€)	284 (51 – 1310)	394 (51 – 10 607)	ns

*RBC* Red blood cell, *PTL* Platelet, *FFP* Fresh frozen plasma, *ns* not significant

Massive transfusion refers to the administration of ten or more red blood cell units. In this cohort, 48 patients (0.002 %) received massive transfusion. Demographic and operative data of these patients is presented in Table 3. The mean age of the massive transfusion patients were 48 years and 33 (69 %) of them were female. Most of the cases (81 %) were related to OC and 72 % of the cases to emergent operations. In addition to RBCs, 78 % of the patients received fresh frozen plasma products (FFP or Octoplas®) and 46 % PLTs. The massive transfusions were associated with marked in-hospital mortality (15 %).

**Table 3:** Demographic and operative data, and the use of other blood component products in patients who were administered massive RBC transfusion (≥10 units)

	Patients with massive RBC transfusion
	*n* = 48 (%)
Males/females	33/15 (69/31)
Mean age ± SD (range)	48 ± 16 (34 – 90)
ASA^a^ I	2 (4.2)
II	7 (15)
III	13 (27)
IV	14 (29)
V	7 (15)
OC/LC	39/9 (81/19)
Elective/emergency^b^	13/34 (28/72)
Mean operative time ± SD (min)	122 ± 74
Intraoperative cholangiography	8 (17)
Common bile duct exploration	6 (13)
Proportion of patients with PTL transfusion	22 (46)
Proportion of patients with FFP transfusion	30 (63)
Proportion of patients with Octaplas® transfusion	7 (15)
In-hospital mortality	7 (15)
Length of hospital stay ± SD (d)	23 ± 14

*ASA* American Society of Anaesthesiologists, *SD* Standard deviation, *RBC* Red blood cell, *PTL* Platelet, *FFP* Fresh frozen plasma

^a^Data regarding ASA class missing from five patients

^b^Data missing from one patient

## Discussion

According to the present study, OC is associated with higher transfusion rate of blood components than LC. In the current data, 13 % of the OC patients and 1.3 % of LC patients received RBC transfusion. Also, for the other blood component products (PLTs, FFP and Octaplas®), the transfusion rates were significantly higher in the OC group. In addition to more invasive nature of OC, this may be partly due to the fact that the OC patients were older and thus more likely to receive anticoagulant therapy. The OC patients also underwent emergent operation more often than LC patients.

The lack of systemic classification of bleeding complications in LC makes the comparison of the results of the current study to the existing body of literature challenging. Some authors have assessed and reported only major vascular injuries (usually including injuries to the aorta and its main branches, vena cava and the portal vein), while life-threatening bleeding may also occur from the liver bed [14]. Vascular injuries may also have been reported as trocar injuries. Other authors have documented bleeding requiring either transfusion or reoperation or less serious intraoperative and postoperative bleeding. Intraoperative and postoperative bleeding may have been further divided into internal (peritoneal cavity of retroperitoneal space) and external (abdominal wall) bleeding based on the localization.

The incidence of postoperative intra-abdominal bleeding has been reported to be 0.69–1.05 % in LC patients [9, 16]. In the analysis of 10 174 LCs by Z’graggen and co-workers [9], bleeding was also the most frequent intraoperative complication occurring in 1,97 % of the cases. In a Finnish series of 1581 LCs, incidence of all bleeding complications was 1.1 % [22] and bleeding complications requiring reoperation occurred in 0.5 % of the cases [23]. Roslyn and co-workers [4] reported the overall incidence of bleeding complications of 0.4 % in the analysis of 42 474 OCs. In their series, intraoperative bleeding was also associated with a significant risk of death. In the current study, the data on massive transfusions indicates, that major bleeding remains a rare but serious complication of cholecystectomy with significant associated mortality. New advantages of technology, such as ultrasonic dissection and anticoagulant pads may decrease the bleeding complications in future register studies.

Previous studies have hardly reported the need of blood transfusion related to LCs and OCs. However, few publications reporting transfusion rates for general laparoscopic operations exist. In their analysis of 14 243 general laparoscopic operations (of which 59.4 % were LCs), Schäfer and co-workers [14] reported 33 patients with intraoperative and 63 patients with postoperative bleeding complications requiring blood transfusion. The overall rate of bleeding complications requiring transfusion was 0.7 % in their series, the overall rate of bleeding complications (including minor bleeding such as laceration of minor vessels) being 4.1 %. Opitz and co-workers [15] reported an overall bleeding rate of 3.3 % in an LC-dominant (52 %) sample of 43 028 of general laparoscopic operations. In their study, the higher transfusion rate (24 %) was observed in patients with postoperative bleeding compared to patients with intraoperative bleeding (7 %, *p* < 0.0001).

Transfusion rates for LC in this study, 1.3 % RBCs and 1.6 % for all blood component products, are higher than reported for above mentioned LC-dominant general laparoscopy samples [14, 15]. About 30 % of patients in these two laparoscopy samples underwent herniotomy or appendectomy, both procedures that do not involve the dissection of the liver bed, a potent source of bleeding. This may explain in part the higher observed transfusion rate in this study. In addition, a previous report shows that the rate of RBC usage in Finland has been rather high compared to that in other European countries partly, because the sufficient blood supply has not limited the availability of blood component products and because of the low risk for transfusion-transmitted viral infections in Finland [19].

Bleeding data of our transfusion register and particular platelet transfusions in the patients undergoing general surgery has been published earlier [20]. One-fourth (27.1 %) of the surgery-related platelet transfusions went to patients who had alimentary tract operations, 11 % in orthopedic surgery, but mainly to patients undergoing cardiac operations. Surgery-related bleeding complications and platelet transfusions occurred most frequently between the age groups 50 and 79 years, and more often in males than females [20]. In obstetric procedures, platelets were used in 267/17916 (1.5 %) operations.

There are several limitations in this study. One is the register-based nature of this study. First, the current data covers only the transfusions associated with the hospital stay during which the cholecystectomy took place, and thus cases of delayed postoperative bleeding requiring transfusion may have been missed. Second, reoperations are not reported. Major bleeding is often defined as a bleeding complication requiring transfusion or a reoperation. However, there was a substantial number of missing diagnosis codes for 60-day reoperations in the data. Combined with the lack of uniform practice for diagnosis and procedure code entries, when a complication occurs, it would have been highly biased to report the rate of reoperations, especially those performed because of bleeding. Third, conversions could not be identified from the data due to the lack of a separate procedure code for conversion in NCSP. Consequently, cases of LC converted into OC are included in the OC group in this study. Since bleeding is a frequent reason for conversion [9, 10, 16, 17], conversions would have been deserved to be analyzed as a group of its own.

In addition, because of the register-based nature of this study, patient-specific predisposing factors for bleeding complications, such as anticoagulant or anti-platelet therapy or liver cirrhosis, could not be identified from the available data. A high incidence of post-operative bleeding has been reported in patients on long-term anticoagulant therapy undergoing LC, even when the anticoagulant therapy was discontinued long enough for the international normalized ratio to be normalized [24]. Additionally, in a Swedish register study, systemic thromboprophylaxis increased the risk of bleeding complications in LC, but the incidence of thromboembolic complication was not significantly reduced [25]. On the other hand, the association between anti-platelet therapy and bleeding complications is controversial, especially in the case of emergency surgery. In a recent retrospective case–control study, long-term aspirin anti-platelet therapy was not associated with increased risk of bleeding complications in emergent LC for acute cholecystitis [26]. Based on the results, the authors concluded that long-term aspirin use should not be used as an independent factor to delay an emergent LC. The impact of the new non-vitamin K antagonist oral anticoagulants on the incidence of bleeding complications associated with LC remains an interesting topic for future research.

## Conclusions

The present study shows that, in additions to other virtues, LC is associated with lower transfusion rates of RBCs, PLTs and FFP products compared to OC. The mean transfused dose for RBCs, PLTs or Octaplas® and the mean cost of transfused blood component products per transfused patient did not differ significantly between LCs and OCs indicating the severity of bleeding complication may not vary substantially between OC and LC. Nevertheless, the observed higher transfusion rate in OC increases the average costs of OC compared to LC.
